# Physical, chemical, and biological control of black rot of brassicaceae vegetables: A review

**DOI:** 10.3389/fmicb.2022.1023826

**Published:** 2022-11-23

**Authors:** Zeci Liu, Huiping Wang, Jie Wang, Jian Lv, Bojie Xie, Shilei Luo, Shuya Wang, Bo Zhang, Zhaozhuang Li, Zhibin Yue, Jihua Yu

**Affiliations:** ^1^College of Horticulture, Gansu Agriculture University, Lanzhou, China; ^2^Institute of Biology, Gansu Academy of Sciences, Lanzhou, China

**Keywords:** cruciferous, black rot, *Xanthomonas campestris* pv. *campestris*, antibacterial activity, disease resistance

## Abstract

As one of the important sources of human nutrition, Brassicaceae vegetables are widely grown worldwide. Black rot caused by *Xanthomonas campestris* pv. *campestris* (*Xcc*) seriously affects the quality and yield of Brassicaceae vegetables. Therefore, it is important to study control methods of *Xcc* for Brassicaceae vegetable production. This paper reviews the physical, chemical, and biological control methods of *Xcc* in Brassicaceae vegetables developed in recent years, and the underlying mechanisms of control methods are also discussed. Based on our current knowledge, future research directions for *Xcc* control are also proposed. This review also provides a reference basis for the control of *Xcc* in the field cultivation of Brassicaceae vegetables.

## Introduction

The Brassicaceae comprises approximately of 3,700 species, including vegetable, forage, and oil-seed brassicas. Moreover, the planting area of Brassicaceae vegetables is expanding rapidly year by year. However, Brassicaceae vegetables are susceptible to infection by numerous fungal and bacterial pathogens, including *Xanthomonas campestris* pv. *campestris* (*Xcc*) (Li et al., 2019). *Xcc* causes black rot (BR) and is one of the most destructive and yield-limiting diseases of Brassicaceae vegetables (Vicente and Holub, 2013; Kong et al., 2021; Koroleva et al., 2021). Since BR was discovered in 1894 in the USA, it has spread rapidly around the world and is now found in major Brassicaceae vegetable producing areas (Garman, 1894; Jensen et al., 2010). Seedborne *Xcc* can survive in crop debris or crucifer weeds, and it is especially damaging to Brassicaceae vegetables. Under conditions with average temperatures between 25–30°C and with sufficient rainfall or heavy dew, *Xcc* enters the host plant vascular system through hydathodes or wounds caused by machinery or insects (Vicente and Holub, 2013). Except for some cases in which no symptoms occur (latent infection), the bacterium causes a systemic vascular disease, the typical symptoms of which are vein blackening, leaf tissue necrosis, and V-shaped chlorotic lesions (Marroni and Germani, 2014). However, symptoms of BR may differ among different Brassicaceae vegetables. These symptoms reduce the quality and value of the Brassicaceae, a crop in which the leaves are the major commercial product (Vicente and Holub, 2013). Indeed, in some cases, Brassicaceae crops can be entirely lost to BR (Jensen et al., 2005). Hence, it is important to study the methods and mechanisms of BR resistance of Brassicaceae vegetables.

Several agricultural practices and culture methods, such as planting material free of *Xcc* (seeds or transplants), crop rotation, and eliminating other possible sources of inoculum, such as residues of infected crops and Brassicaceae weeds, have been reported to control BR (Krauthausen et al., 2011; Ryan et al., 2011; Barzman et al., 2015). However, these methods are all preventative, and cannot control BR once the plant is infected (Vicente and Holub, 2013; Holtappels et al., 2021). Planting BR resistant varieties is the most economical and effective approach to control this disease. Although some Brassicaceae, such as radish (*Raphanus sativus*) and Chinese cabbage (*Brassica rapa*), BR resistant varieties have been developed, the vast majority of Brassicaceae vegetables still lack effective BR resistant varieties. Unfortunately, the large number of physiological races of *Xcc* and the differences in the genome types of Brassicaceae make it difficult to cultivate BR resistant varieties of cabbage and other vegetables. At present, *Xcc* isolates have been differentiated into 11 physiological races based on comparisons of their whole genome sequences, and physiological races 1 and 4 are the two main races that damage Brassicaceae vegetables (Vicente et al., 2001; Qian et al., 2005; Vicente and Holub, 2013; Singh et al., 2016; Cruz et al., 2017). From a production point of view, cultivating varieties resistant to race 1 and race 4 is the main direction of breeding for resistance to BR. However, sources resistant to race 1 and 4 are very rare in genome C (*Brassica oleracea*), while they are relatively common in A and B genomes (*B. rapa* and *Brassica nigra*), which has seriously restricted the breeding of BR resistance varieties of cabbage and other genome C-type plants (Afrin et al., 2018). Therefore, the study of other control methods, especially highly efficient broad-spectrum and pollution-free control methods, has important production guiding value for Brassicaceae vegetable production (Lamichhane et al., 2017).

A large number of *in vivo* and *in vitro* studies have shown that there are many physical methods, chemical treatments, and biological control methods that can inhibit the activity or pathogenicity of *Xcc*. These studies provide references for the pollution-free control of Brassicaceae vegetables BR. However, there is no review of the physical (hot water, ultraviolet light), chemical (pesticides, chemical substances), and biological (plant aqueous extracts, *Xcc* antagonistic bacteria, biological products) methods for *Xcc* control. The purpose of the present review is to provide a theoretical basis for the control of BR in the production of Brassicaceae vegetables by analyzing reported control methods and control mechanisms, and to predict future potential control methods for *Xcc*.

## Physical control of *Xanthomonas campestris* pv. *campestris*

### Hot water

As a common and effective strategy that is widely used in seed disinfection in production, soaking seeds in 45–55°C hot water can economically and effectively kill microbes that are latent or stuck to the inside and outside of seeds. As a bacterium, *Xcc* can spread over long distances via seeds. The usual lethal temperature of *Xcc* is 40°C; therefore, *Xcc* in seeds can be killed by hot water treatment at higher temperatures. Hot water was first observed to kill the *Xcc* in the 1920s (Walker, 1923). After 50 or 52°C hot water treatment of cauliflower (*Brassica oleracea* L. var. *botrytis* L.) seeds, the incidence of BR in the field decreased significantly (Sharma, 1981). Soaking in 52°C hot water for 0.5 h had the best control effect (Shah et al., 1985). Subsequent 3-year and 1-year trials also showed that hot water treatment (50°C for 20 min) of cauliflower (*B. oleracea* L. var. *botrytis* L.) seeds decreased *Xcc* activity (Shah et al., 1985; Van Der Wolf and Van Der Zouwen, 2010). In addition to killing *Xcc* in the seeds, the process of soaking allows dry seeds to quickly absorb the water needed for germination. However, soaking seeds in hot water not only had a control effect on *Xcc*, but also strongly decreased the germination rate of treated Chinese cabbage (*B. rapa*) seeds (Velasco et al., 2013). It is possible that the temperature of the water used for soaking was too high or the soaking time was too long, resulting in seed damage.

### Nanoparticles

As a high-tech invention in the 21st century, nanoparticles have been widely used in agricultural production, plant protection, plant nutrition, and other fields. For *Xcc* control, 800 mg/L Gly-Cu(OH)_2_ nanoparticles (mean diameter of 240 nm) increased the *Xcc* resistance of Chinese cabbage (*B. rapa* pekinensis), and had a better effect than 400–800 mg/L of Kocide, a fungicide/bactericide (Dong et al., 2020). In addition to nano-copper, nano-silver has also been found to prevent *Xcc* infection. The growth of *Xcc* could be inhibited at 0.02 μg/ml, and >0.1 μg/ml nano-silver nanoparticles (AgNPs) could destroy the *Xcc* cell membrane (Gan et al., 2010). The antibacterial effect of AgNPs depended on their size and concentration (Velasco et al., 2013; Pecenka et al., 2021). Nanoparticles can replace the hydrogen bonds between thymidine and purine, and cytosine and guanine in the double helix structure of DNA by combining with DNA in the cell membrane and cell wall of bacteria, resulting in changes to the molecular structure of bacterial DNA, inhibiting the synthesis of DNA, RNA, and proteins, thereby inactivating pathogenic bacteria (Duran et al., 2010).

### Other physical methods

In addition to soaking seeds and using nanoparticles, other physical methods can also be used in *Xcc* control. Using different ultraviolet light (UV-C) doses (1.3–7.5 kJ/m^2^), study have showed that low doses of UV-C treatment of seeds could elicit resistance to *Xcc* and improve the quality and growth of cabbage (*Brassica oleracea* var. *capitata*). UV-C at 3.6 kJ/m^2^ was effective in reducing BR and the population density of *Xcc* in infected cabbage leaves (Brown et al., 2001). UV-C can change the genetic material of pathogenic bacteria and change cellular transcription characteristics, such that the organism loses the ability to synthesize proteins and its reproduction ability (Hamkalo, 1972). In addition to UV-C pre-treatment before sowing, because *Xcc* enters the plant through wounds or/and the hydathodes on the leaves, BR can also be controlled by spraying the agents to form a protective film on the leaves during the growth period. *Xcc* infection is particularly harmful because of the formation of biofilms, which contain degradative extracellular enzymes and other virulence factors (Dow et al., 2003). By forming an insulating layer on cabbage (*B. oleracea* var. *capitata*) leaves, a novel carbon-based biomaterial, Se/C (formed by adding with a very small amount of selenium into carbon), could restrain *Xcc* infection (Cao et al., 2020).

## Chemical control of *Xanthomonas campestris* pv. *campestris*

### Pesticides

Pesticides are widely used to prevent and control plant diseases (Lamichhane et al., 2017). Studies have shown that some pesticides can provide control of *Xcc*. Seed dipping in 100 ppm streptocycline significantly reduced the incidence of BR on cauliflower (*B. oleracea* L. var. *botrytis* L.) by seed dipping (Sharma, 1981). The 0.1% streptocycline seed treatment had a good control effect on *Xcc* in a 3-year cauliflower trial (Shah et al., 1985). By comparing the *Xcc* control efficiency of streptomycin, oxytetracycline, chloramphenicol, rifampicin, and mancozeb at 200 ppm, it was found that streptomycin provided 100% BR control in cauliflower, followed by oxytetracycline and chloramphenicol, while mancozeb was ineffective (Lenka and Ram, 1997). In addition, 16 ml/kg Metham-sodium could significantly reduce the counts of the *Xcc* in cauliflower stems, siliques, and seeds, and reduced the incidence of *Xcc* symptoms. Moreover, the effectiveness of control was also related to the depth of the application of the Metham-sodium into the soil (Kritzman and Ben-yephet, 1990). By analyzing the germination rate and disease symptoms of Chinese cabbage seeds artificially inoculated with *Xcc* and soaked in streptocycline and 18 other pesticides, it was observed that the anti-*Xcc* effect of 200 g/ml streptocycline was higher than that of mercuric chloride, Mancozeb, carbendazim, 2-methoxyethylmercury chloride, and ampicillin; while the anti-*Xcc* effects of chlortetracycline, oxytetracycline, and chloramphenicol were higher than that of streptocycline at 200 g/ml (Bhat and Masoodi, 2000). Similarly, studies on cabbage (*B. oleracea* var. *capitata*) and kale (*Brassica oleracea* var. *acephala*) showed that pesticides had a good control effect on BR. Kocide and Actigard significantly suppressed the incidence of BR on cabbage (Langston and Cummings, 2003). Lime sulfur could not only prevent BR, but also improved the nutritional quality of kale (*B. oleracea* var. *acephala*) in non-inoculated conditions (Nunez et al., 2018). Although the above studies showed that many chemical pesticides can control BR, the molecular mechanisms of resistance to these agents have not been determined.

By spraying Validamycin-A (VMA) and acibenzolar-S-methyl (ASM) on cabbage (*Brassica oleracea* var. *capitata*) leaves, studies investigated the BR control mechanism of the two agents, and found that 62.5 g/L VMA effectively inhibited the production of *Xcc* extracellular polysaccharide (EPS), and injection of EPS from *Xcc* on medium containing VMA produced fewer lesions, suggesting that VMA might inhibit the multiplication of *Xcc* and affect the quantity and quality of EPS (Ishikawa et al., 2004). Unlike EPS, ASM could decrease *Xcc* symptoms and oxidative stress by increasing the activities of superoxide dismutase, peroxidase, and ascorbate peroxidase (Amaral et al., 2019), while the activities of chitinase, β-1,3-glucanase, and polyphenol oxidase, and the concentrations of hydrogen peroxide and malondialdehyde, decreased (Fontana et al., 2021).

### Chemical substances

#### Soaking the seeds with chemicals

Pretreatment of seeds with chemical substances can also prevent *Xcc* infection. Soaking seeds for 16 h in a 10–20 g/kg calcium hypochlorite slurry reduced *Xcc* undetectable levels in cabbage (*B. oleracea* var. *capitata*) seeds (Schultz et al., 1986). Soaking seeds for 30 min using 3% hydrogen peroxide also killed 100% of *Xcc* in cabbage (*B. oleracea* var. *capitata*) seeds (Sanna et al., 2022). Pre-treatment of *Xcc*-contaminated cabbage (*B. oleracea* var. *capitata*) seeds with CAC-717 (a new disinfectant produced by applying an electric field and water flow to distilled water containing calcium hydrogen carbonate to produce mesoscopic crystals) significantly reduced *Xcc* cell numbers recovered from the seeds compared with using distilled water (Sakudo et al., 2020).

#### CuSO_4_

CuSO_4_ is widely used in disease control because of its low cost and broad-spectrum antimicrobial activities (Chen et al., 2021). The first study of CuSO_4_ on *Xcc* showed that CuSO_4_ was ineffective in *Xcc* control (Lenka and Ram, 1997). However, the *Xcc* killing effects of t-butyl hydroperoxide and hydrogen peroxide increased after the addition of 100 mM CuSO_4_ to Silva-Buddenhagen medium (Patikarnmonthon et al., 2010). The incidence of BR in kale (*B. oleracea* var. *acephala*) was also reduced after Bordeaux mixture (an inorganic copper bactericide mainly composed of CuSO_4_) was applied, and the nutritional value also improved in non-inoculated kale (*B. oleracea* var. *acephala*) (Nunez et al., 2018). Cu(OH)_2_ has also been found to improve broccoli (*B. oleracea* L. var. *botrytis* L.) BR resistance (Krauthausen et al., 2011). The above studies indicated that Cu^2+^ was the main factor that increased *Xcc* resistance, in which Cu^2+^ kills bacteria mainly by denaturing and solidifying bacterial proteins (Cooksey, 1990).

#### Other chemical substances

In addition to the use of sterilizing chemicals for seed immersion disinfection, chemical treatment during the growing period can also enhance plant resistance to *Xcc* invasion. After spraying benzoic acid, the BR resistance of cauliflower (*B. oleracea* L. var. *botrytis* L.) increased (Krauthausen et al., 2011). Foliar spraying of p-coumaric acid (pCA) promoted the accumulation of specific hydroxycinnamic acids, pCA, ferulic acid, sinapic acid epigallocatechin and epigallocatechinin in *Xcc*-inoculated Chinese cabbage (*B. rapa*) (Islam et al., 2018). pCA primed the JA-signaling mediated induction of phenylpropanoid biosynthesis to produce *Xcc* resistance in oilseed rape (*Brassica napus*), in which the expression of phenylpropanoid biosynthesis-related genes was upregulated during pre-treatment with pCA (Islam et al., 2019). Using 10 kinds of epigenetic modulating chemicals (Azacytidine, -Oryzanol, Lomeguatrib, RG108, Zebularine, Cambinol, CAY10602, Sirtinol, SRT1720 Hydrochloride, Suramine), Baranek et al. found the use of DNA demethylating chemicals unambiguously caused a durable decrease in *Xcc* virulence via altered sirtuin activity, even after its re-isolation from infected Chinese cabbage (*B. rapa*) (Baranek et al., 2021). Xantho-oligosaccharides not only inhibited the growth of *Xcc*, but also reduced the production of xanthan, which is associated with the virulence of *Xcc* (Qian et al., 2006). These studies suggested that the incidence of BR could be reduced by application chemical substances that enhance the phenylpropane synthesis pathway in plants or reduce *Xcc* toxicity.

## Biological control of *Xanthomonas campestris* pv. *campestris*

### Aqueous extracts

Many chemical substances and pesticides are expensive and harmful to the environment, thus it is important to explore environmentally friendly BR control methods for pollution-free Brassicaceae vegetable production (Adesola et al., 2021; Mouna et al., 2021). Therefore, mostly in laboratory studies, plant extracts can be explored as candidates for the management of BR. Studies have shown that extracts from many plants have killing effects on *Xcc* in plants and/or growth on culture medium. Tiwari et al. found that *Datura metel*, *Allium sativum*, *Zingiber officinale*, *Parthenium hysterophorus*, and *Spiranthus indicus* could kill *Xcc* (Tiwari et al., 2004). And 250–1,000 mg/L of *Mikania glomerata* alcoholic extract could inhibit *Xcc* growth *in vitro* (Vigoschultz et al., 2006). Furthermore, 0.5 and 0.1 mg/ml of methanolic, hydroalcoholic, and hydroalcoholic maltodextrin *M. glomerata* extracts showed bacteriostatic and bactericidal effects by altering the membrane permeability and biofilm formation of *Xcc* (Fontana et al., 2021). Using *in vitro* and *in vivo* tests of 20 kinds of botanical extracts, it was concluded that the extracts of *A. sativum*, *Azadirachta indica*, *Tamarix aphylla*, *Vernonia anthelmentica*, *Plumbago zelanicum*, and *Tegetes erecta* significantly suppressed the growth of *Xcc* and resulted in better seed germination and plant vigor (Sain, 2007). Research on the bacteriostatic activity of 20 kinds of botanical extracts also showed that the leaf extracts of *Acacia arabicae*, *Acacia fernesiana*, *Acacia catechu*, *Achyranthus asper*, *Aegle marmelos*, *Asparagus racemosus*, *A. indica*, *Callistemon lanceolatus*, and *Camellia sinensis* showed an inhibitory effect against *Xcc* (Bhardwaj and Laura, 2009). *Xcc* was also kill by the petal extracts of *T. erecta*, *Chrysanthemum coronarium*, *A. fernesiana*, *Anthocephalus cadamba*, *Bombax malabaricum*, *Lathyrus odoratus*, *Rosa damascena*, and *Thevetia nerifolia* (Bhardwaj and Bhardwaj, 2011). These studies indicated that the anti*-Xcc* activity was not only related to the plant material, but also to the plant parts. Meanwhile, extracts from the same plant using different solvents also had different antibacterial properties. The water extracts of *Ocimum gratissimum* and *Tylophora asthmatica* were effective in inhibiting the growth of *Xcc*, while the alcohol extract of *O. gratissimum* was the most effective in inhibiting the growth of *Xcc*, followed by that of *Calotropis gigentea*, *T. asthmatica*, *Ocimum sanctum*, *Nigella sativa*, and *Ruta graveolens* (Kumar et al., 2018). In addition, the *Trichoderma atroviride* metabolite, 6-pentyl-α-pyrone, also could inhibit *Xcc* by increasing the antibiofilm activity (Papaianni et al., 2020). Most of these studies only analyzed the antibacterial activity of the total extracts from the whole plant or some organs and did not study the components related to BR resistance (Table 1). The resistant secondary metabolites such as flavonoids, phenolic acids, alkaloids, isothiocyanates, tannins, and saponins in these plants might be the components of *Xcc* resistance.

**TABLE 1:** Effects of extracts from different crops and different tissues on the antibacterial ability of *Xcc*.

Plant species	Part used	Solvent	Concentration	Zone of minimum inhibition (mm)*	References
*Acacia arabicae*	Bark	Water	15 g petals/100 ml water	18.0 ± 1.24	Bhardwaj and Laura, 2009
*Acacia catechu*	Bark	Water	15 g petals/100 ml water	16.5 ± 2.15	Bhardwaj and Laura, 2009
*Acacia fernesiana*	Seed	Water	15 g petals/100 ml water	10.0 ± 1.78	Bhardwaj and Laura, 2009
	Petal	Water	15 g petals/100 ml water	11.50 ± 1.24	Bhardwaj and Bhardwaj, 2011
*Achyranthus asper*	Stem	Water	15 g petals/100 ml water	16.5 ± 1.88	Bhardwaj and Laura, 2009
*Aegle marmelos*	Fruit	Water	15 g petals/100 ml water	17.5 ± 1.13	Bhardwaj and Laura, 2009
*Allium sativum*	Rhizomes	Water	Air-dried rhizome: water = 1:1 (W/V)	9.45–24.87; –	Sain, 2007; Tiwari et al., 2004
*Anthocephalus cadamba*	Petal	Water	15 g petals/100 ml water	10.50 ± 2.15	Bhardwaj and Bhardwaj, 2011
*Asparagus racemosus*	Root	Water	15 g petals/100 ml water	16.5 ± 1.16	Bhardwaj and Laura, 2009
*Azadirachta indica*	Leaf	Water	Air-dried leaf: water = 1:1 (W/V); 15 g petals/100 ml water	7.99–23.75;16.0 ± 0.84	Sain, 2007; Bhardwaj and Laura, 2009
*Bombax malabaricum*	Petal	Water	15 g petals/100 ml water	11.00 ± 1.78	Bhardwaj and Bhardwaj, 2011
*Callistemon lanceolatus*	Bark	Water	15 g petals/100 ml water	14.5 ± 1.46	Bhardwaj and Laura, 2009
*Camellia sinensis*	Leaf	Water	15 g petals/100 ml water	19.5 ± 1.25	Bhardwaj and Laura, 2009
*Chrysanthemum coronarium*	Petal	Water	15 g petals/100 ml water	23.50 ± 0.35	Bhardwaj and Bhardwaj, 2011
*Datura metel*	Leaf	Water	Air-dried leaf: water = 1:1 (W/V)	–	Tiwari et al., 2004
*Lathyrus odoratus L.*	Petal	Water	15 g petals/100 ml water	10.50 ± 0.84	Bhardwaj and Bhardwaj, 2011
*Mikania glomerata*	Whole plant	Alcoholic	250–1,000 mg/L	–	Vigoschultz et al., 2006
	Leaf	Methanolic, hydroalcoholic, hydroalcoholic maltodextrin	0.5, 0.5, 0.1 mg/L	–	Fontana et al., 2021
*Ocimum gratissimum*	Leaf	Water, alcohol	20 g Leaf/50 ml 70% alcohol	29.33, 31.66	Kumar et al., 2018
*Parthenium hysterophorus*	Flower	Water	Air-dried flower: water = 1:1 (W/V)	–	Tiwari et al., 2004
*Plumbago zelanicum*	Leaf	Water	Air-dried leaf: water = 1:1 (W/V)	7.35–20.42	Sain, 2007
*Rosa damascena*	Petal	Water	15 g petals/100 ml water	13.50 ± 2.47	Bhardwaj and Bhardwaj, 2011
*Spiranthus indicus*	Flower	Water	Air-dried flower: water = 1:1 (W/V)	–	Tiwari et al., 2004
*Tagetes erecta*	Petal	Water	15 g petals/100 ml water	24.0 ± 0.26	Bhardwaj and Bhardwaj, 2011
*Tamarix aphylla*	Leaf	Water	Air-dried leaf: water = 1:1 (W/V)	20.46	Sain, 2007
*Tegetes erecta*	Bulb	Water	Air-dried bulb: water = 1:1 (W/V)	6.34–20.46	Sain, 2007
*Thevetia nerifolia*	Petal	Water	15 g petals/100 ml water	11.00 ± 2.25	Bhardwaj and Bhardwaj, 2011
*Tylophora asthmatica*	Leaf	Water, alcohol	20 g Leaf/50 ml 70% alcohol	24.33, 25.33	Kumar et al., 2018
*Vernonia anthelmentica*	Bulb	Water	Air-dried bulb: water = 1:1 (W/V)	6.98–22.83	Sain, 2007
*Zingiber officinale*	Rhizomes	Water	Air-dried rhizomes: water = 1:1 (W/V)	–	Tiwari et al., 2004

*Means items are not mentioned in the references.

In addition to the above medicinal plants and non-Brassicaceae plants, a study on cress (*Lepidium sativum*), salad rocket (*Eruca sativa*), broccoli (*Brassica olerecea* L. var. *italica*), white cabbage (*B. olerecea* L. var. *capitata*), and tronchuda cabbage (*B. oleracea* L. var. tronchuda cv. *Tronchuda*) showed that the hydrolytic products of glucosinolates could inhibit *Xcc* growth, although there were no significant correlations between *Xcc* infection and total phenolics (Aires et al., 2011). However, using the methanolic extracts of Chinese cabbage (*B. rapa*) leaves, it was concluded that the glucosinolates (Gluconapin, and gluconapin-isothiocyanate) and phenolics had inhibitory effects on the development of *Xcc*, and there was a significant positive correlation between their concentration and the inhibitory effect (Velasco et al., 2013). In addition to extracts derived from plants using solvents, *Piper hispidum* leaves essential oils (trans-a-bisabolene, b-pinene, a-pinene, allo-aromadendrene, (-) spatulenol, and L-linalool), obtained by hydrodistillation in a Clevenger-type apparatus, also showed weak antibacterial activity against *Xcc* (Sánchez Pérez et al., 2014). The thymol and carvacrol in *Lippia gracilis* essential oils are the main anti-*Xcc* components (Rafael et al., 2019). Most of these studies only studied the antibacterial activity and BR resistance of relevant plant extracts, and did not study their antibacterial mechanism; however, they also provided a theoretical basis and data support for the subsequent development of plant-derived pesticides for BR control.

### Bacillus

*Bacillus* are gram-positive rhizobacteria with broad-spectrum bactericidal activity, which secrete a variety of enzymes and antibiotics to inhibit the growth of other bacteria and can prevent and control a variety of plant diseases (Babin et al., 2022; Jing et al., 2022). Previous studies identified that there is an antagonistic interaction between *Xcc* and *Bacillus* species in the natural ecosystem. Studies on the resistance of *Bacillus* to *Xcc* mainly include *in vitro* bacteriostatic experiments and resistance mechanism studies on Brassicaceae crops. *In vitro* tests using *Bacillus* isolates found that *Bacillus amyloliquefaciens*, *Bacillus subtilis*, and *Bacillus pumilus* isolates could inhibit the growth of *Xcc* to varying degrees (Sain, 2007; Wulff et al., 2010), with *B*. *amyloliquefaciens* being the most effective (Wulff et al., 2010; Li’aini et al., 2017). *B. subtilis*, *B. pumilus*, *Bacillus megaterium*, *Bacillus cereus*, *Bacillus velezensis*, *Paenibacillus*, and *Bacillus thuringiensis* also showed antibiotic activity against the *Xcc* (Luna et al., 2002; Ghazalibiglar et al., 2016; Liu et al., 2016a; Jelusic et al., 2021). In addition, there were significant correlations between the mean diameter of the inhibition zone, the type of bacteria, and the type of culture medium (Issazadeh et al., 2012; Silva et al., 2018). The gas chromatography-mass spectrometry (GC-MS) and high performance liquid chromatography (HPLC)-electrospray ionization (ESI)-quantitative time of flight (qTOF)/mass spectrometry (MS) results for the benzene extracts and ethyl acetate chemical compositions of two *Bacillus* strains showed that they contained numerous antimicrobial volatile organic compounds (e.g., alkenes, benzenes, carboxylic acids, indoles, and pyrazines), and antimicrobial metabolites (lipopeptides, and/or antibiotics) (Jelusic et al., 2021). Moreover, the biosynthesis of surfactin, kurstakin, bacillomycin, and iturin are involved in the killing process of *Xcc*, and the contents of these antibacterial substances are closely related to resistance to *Xcc* (Wulff et al., 2010; Jelusic et al., 2021; Macha et al., 2021).

*In vivo* tests concluded that the *Bacillus* and *B. amyloliquefaciens* could significantly reduce the incidence and severity of BR in the foliage, stems, and heads of cabbage (*B. oleracea* var. *capitata*) and oilseed rape (*B. napus*) (Sain, 2007; Massomo et al., 2010; Wulff et al., 2010; Li’aini et al., 2017; Jelusic et al., 2021). Not only can *Bacillus* strains prevent *Xcc*, but also their cell-free culture medium exerts a killing effect on *Xcc*. *Paenibacillus*, *B. velezensis*, and *B. megaterium* cell-free supernatants were found to be effective against *Xcc* in cabbage (*B. oleracea* var. *capitata*), kale (*B. oleracea* var. *acephala*), and oilseed rape (*B.napus*) (Liu et al., 2016a; Silva et al., 2018; Jelusic et al., 2021). Besides, the control effect of *Bacillus* is not only related to the species of bacteria, but also to the species of plants and their growing environment. By evaluated the control efficiency of *B. subtilis* against *Xcc* using cabbage (*B. oleracea* var. *capitata*), cauliflower (*B. oleracea* L. var. *botrytis* L.), oilseed rape (*B. napus*) and broccoli (*B. oleracea* L. var. *botrytis* L.) grown in three consecutive growing seasons and on two types of soil, it found that *B. subtilis* was effective in broccoli, but not in cabbage and rape during the main rainy season in clay loam soil, and a limited biological control effect was still observed when these crops were grown in sandy loam soil (Wulff et al., 2002). Moreover, root application had the best effect compared with seed soaking, leaf or foliage (cotyledons) spraying and soil drenching (Massomo et al., 2010). In addition to reducing the incidence of BR, *B. velezensis*, *B. mojavensis*, and *Paenibacillus* could also improve crop growth (shoot fresh weight, shoot dry weight, root fresh weight, and root dry weight) and yield (Sain, 2007; Wulff et al., 2010; Ghazalibiglar et al., 2015, 2016; Liu et al., 2016b). A study on the anti-*Xcc* mechanism of *B. thuringiensis* showed that RpfF is essential for the production of six diffusible signal factor family signals in *Xcc*, which employ the same signaling pathways to regulate their biological functions in *Xc*c and have similar effects on reduction of cell division, sporulation, and antibiotic resistance of *B. thuringiensis*. Abrogation of RpfF decreased the competitive capability of *Xcc* against *B. thuringiensis* on the surface of Chinese cabbage (*B. rapa*) leaves (Deng et al., 2016).

### Pseudomonas

Non-pathogenic *Pseudomonas* is active in the plant rhizosphere, and belongs to the PGPR (Plant Growth-promoting Rhizobacteria). *Pseudomonas* can not only promote plant growth by producing active substances or mineral elements, but also inhibits or hinders the development of pathogenic microorganisms in the root zone by producing metabolic substances or via competition (Muthukumar et al., 2022). Studies have shown that *Pseudomonas*, similar to *Bacillus*, also has a controlling effect on *Xcc*. *In vitro* tests, using different kinds of PGPRs found that *P. aeruginosa* KA19 and AP218 could significantly inhibit the growth of *Xcc* (Mishra and Arora, 2012; Liu et al., 2016a). Moreover, *Pseudomonas orientalis* X2-1P obtained from the oilseed rape (*B. napus*) phyllosphere was also found to be effective against *Xcc in vitro* when applied as a whole culture (Jelusic et al., 2021; Table 2). *In vivo*, *P. aeruginosa* KA19 was effective in reducing *Brassica campestris* BR lesions via foliar spray or by combined seed soaking and soil drenching in the greenhouse (Mishra and Arora, 2012). The application of *P. orientalis* X2-1P cell-free supernatant was also found to be effective against *Xcc* (Jelusic et al., 2021). In addition, *Pseudomonas fluorescens* can induce *Xcc* resistance in cabbage (*B. oleracea* var. *capitata*) seedlings (Umesha and Roohie, 2017). GC-MS and HPLC-MS analyses indicated that there were numerous antimicrobial volatile organic compounds (e.g., alkenes, benzenes, carboxylic acids, indoles, and pyrazines, etc.), lipopeptides, and/or antibiotics in *P. orientalis* ethyl acetate and benzene extracts (Jelusic et al., 2021; Table 3).

**TABLE 2:** Positive antibacterial effects of bacterial strains on *Xcc*.

Strains types	Strains species	References
*Bacillus*	*B. amyloliquefaciens*	Wulff et al., 2010; Li’aini et al., 2017
	*B. pumilus*	Luna et al., 2002; Wulff et al., 2010; Issazadeh et al., 2012
	*B. cereus*	Issazadeh et al., 2012
	*B. megaterium pv. cerealis*	Luna et al., 2002; Issazadeh et al., 2012; Jelusic et al., 2021
	*B. subtilis*	Luna et al., 2002; Wulff et al., 2010; Issazadeh et al., 2012
	*B. thuringiensis*	Luna et al., 2002; Issazadeh et al., 2012; Mishra and Arora, 2012
	*B. velezensis*	Liu et al., 2016a; Jelusic et al., 2021; Macha et al., 2021
*Pseudomonas*	*Pseudomonas aeruginosa*	Mishra and Arora, 2012; Liu et al., 2016a
	*Pseudomonas orientalis*	Jelusic et al., 2021
*Bacteriophage*	Bacteriophage	Marroni and Germani, 2014
	Bacteriophage	Nagai et al., 2017

**TABLE 3:** Effects of different strains on *Xcc* resistance.

Strains types	Strains species	Plant species	References
***Bacillus***	*Bacillus*	*Brassica oleracea* var. *capitata*	Massomo et al., 2010; Wulff et al., 2010
	*Bacillus*	*Brassica oleracea* L. var. *botrytis* L.	Sain, 2007; Massomo et al., 2010; Mishra and Arora, 2012
	*B. amyloliquefaciens*	*Brassica oleracea* var. *capitata*	Li’aini et al., 2017
	*B. megaterium*	*Brassica napus*	Liu et al., 2016a; Jelusic et al., 2021
	*B. mojavensis*	*Brassica rapa*	Liu et al., 2016b
	*B. subtilis*	*Brassica oleracea* var. *capitata*	Wulff et al., 2002
	*B. subtilis*	*Brassica oleracea* L. var. *botrytis* L.	Wulff et al., 2002
	*B. subtilis*	*Brassica napus*	Wulff et al., 2002
	*B. subtilis*	*Brassica oleracea* L. var. *botrytis* L.	Wulff et al., 2002
	*B. subtilis*	*Brassica oleracea* var. *capitata*	Wulff et al., 2002
	*B. subtilis*	*Brassica napus*	Wulff et al., 2002
	*B. thuringiensis*	*Brassica rapa*	Deng et al., 2016
	*B. velezensis*	*Brassica napus*	Jelusic et al., 2021
	*B. velezensis*	*Brassica rapa*	Liu et al., 2016b
	*Paenibacillus*	*Brassica oleracea* var. *acephala*	Ghazalibiglar et al., 2015; Ghazalibiglar et al., 2016; Silva et al., 2018
	*Paenibacillus*	*Brassica oleracea* var. *capitata*	Ghazalibiglar et al., 2015, 2016; Liu et al., 2016b
Bacteriophage	Bacteriophage	*Brassica oleracea var. capitata*	Marroni and Germani, 2014; Ha et al., 2017; Schaad and Ignatov, 2019
	Bacteriophage	*Brassica oleracea* L. var. *botrytis* L.	Ha et al., 2017; Nagai et al., 2017
	Bacteriophage	*Brassica campestris*	Schaad and Ignatov, 2019
	Bacteriophage	*Brassica nigra*	Schaad and Ignatov, 2019
	Bacteriophage	*Brassica geniculata*	Schaad and Ignatov, 2019
	Bacteriophage	*Raphanus sativus*	Schaad and Ignatov, 2019
*Pseudomonas*	*Pseudomonas aeruginosa*	*Brassica campestris*	Mishra and Arora, 2012; Liu et al., 2016a
	*Pseudomonas fluorescens*	*Brassica oleracea var. capitata*	Umesha and Roohie, 2017
	*Pseudomonas orientalis*	*Brassica napus*	Jelusic et al., 2021
Other strains	*Acinetobacter lactucae* QL-1	*Raphanus sativus*	Ye et al., 2019a
	*Burkholderia anthina* HN-8	*Brassica rapa*	Ye et al., 2019b
	*Cupriavidus* HN-2	*Raphanus sativus*	Ye et al., 2019b

### Bacteriophages

Bacteriophages are viruses that infect bacteria universally in the environment (Fischetti, 2008). They can not only “obligate hunt” and “precision target” the pathogenic bacteria in soil, reducing their survival competitiveness, but also can adjust the structure of the rhizosphere soil flora, restore community diversity, and increase the abundance of beneficial bacteria in the community (Da Silva et al., 2018; Holtappels et al., 2021). The initial use of bacteriophages to control plant disease dates back almost a century: they were first used to control *Xcc* in 1924 (Mallmann and Hemstreet, 1924). Bacteriophages significantly reduced the concentration of *Xcc* in infected seed extracts, and the disease rate after bacteriophage treatment was reduced by 2.0–4.1 times in cabbage (*B. oleracea* var. *capitata*) seedlings compared with that in the control (Ha et al., 2017; Holtappels et al., 2022). A bacteriophage suspension was also effective in controlling *Xcc* in cabbage (*B. oleracea* var. *capitata*) and cauliflower (*B. oleracea* L. var. *botrytis* L.) (Marroni and Germani, 2014; Nagai et al., 2017). Considering the different pathogenicities of *Xcc* in different physiological races, the BR could control by releasing weakly virulent *Xcc*. Potentially, weakly virulent *Xcc* could be deployed to activate defense mechanisms and increase the persistence of bacteriophages specific to the target *Xcc* on *Brassica campestris*, *B. nigra*, *Brassica geniculata*, and *R. sativus* leaf surfaces (Schaad and Ignatov, 2019). A study on the anti-*Xcc* mechanism showed that the *Xcc*-specific bacteriophage *Xcc*φ1 could inhibit *Xcc* by interfering with the genes (*rpf*, *gumB*, *clp*, and *manA*) involved in the formation of biofilms (Papaianni et al., 2020)

### Other bacteria

Studies have shown that in addition to *Bacillus*, *Pseudomonas*, and bacteriophages, certain other bacteria can also inhibit or kill *Xcc*. *Acinetobacter lactucae* QL-1 effectively attenuated *Xcc* virulence through quorum quenching in radish (*R. sativus*), and co-inoculation of *Xcc* and QL-1 significantly reduced the BR incidence and severity (Ye et al., 2019a). Using two novel diffusible signal factor degrading strains, *Cupriavidus* HN-2 and *Burkholderia anthina* HN-8, which were isolated from contaminated soil. It was found that HN-2 and HN-8 could substantially reduce BR disease severity caused by *Xcc* in radish (*R. sativus*) and Chinese cabbage (*B. rapa*) (Ye et al., 2019b,^2020^; Table 3).

### Other biological control methods

In addition to plant extracts and *Xcc* antagonism, biological products can also help resist *Xcc* by improving plant resistance. The application of biofertilizer formed by the fermentation of cow manure and sucrose molasse reduced kale (*B. oleracea* var. *acephala*) BR severity by 56% in the field, and increased phenylalanine ammonia lyase, catalase, and peroxidase activities and the lignin content (Nunez et al., 2018). Hence, application of biofertilizer is a promising technique to control BR of Brassicaceae vegetables and also improves their nutritional quality and yield. Milk-based products (raw milk and whey) also reduced kale (*B. oleracea* var. *acephala*) BR severity by 44% in the field; meanwhile, the antioxidant activity, crude protein, and fiber contents increased after spraying raw milk on the *Xcc*-inoculated leaves (Nunez et al., 2018).

## Future work

Black rot (caused by *Xcc*) is one of the most important diseases of Brassicaceae vegetables worldwide, which seriously threatens the production of Brassicaceae vegetables and causes significant economic losses to growers (Cho et al., 2012; Kuznetsov et al., 2020; Pang et al., 2020). Research on the resistance methods and mechanisms of BR are important for the production of Brassicaceae vegetables. To control BR, researchers and growers have studied disease resistance breeding, disease resistance methods, and disease resistance mechanisms (Ryan et al., 2011; Barzman et al., 2015). However, because of the large number of *Xcc* physiological races and the diversity of Brassicaceae vegetable genome types, currently, BR resistant materials are relatively rare, and lack practicality and diversity (Vicente et al., 2001; Vicente and Holub, 2013; Singh et al., 2016; Cruz et al., 2017). Therefore, there is an urgent need to study the disease resistance methods and mechanism of Brassicaceae vegetables.

**FIGURE 1:**
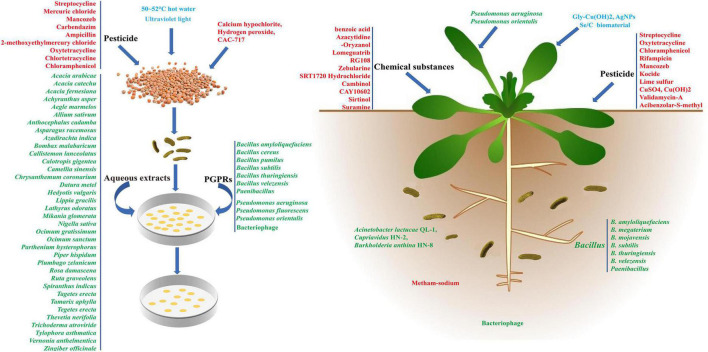
The control process of *Xanthomonas campestris* pv. *campestris* (*Xcc*) by different physical, chemical, biological methods and related agents. The blue, red, and green fonts represent different physical, chemical, and biological *Xcc* prevention methods, respectively.

Similar to most other plant disease control methods, BR control is mainly achieved by improving plant resistance and reducing the pathogenicity of *Xcc*. At present, a variety of antibiotics and pesticides, such as streptocycline, oxytetracycline, and chloramphenicol have been screened using *in vitro* antibacterial tests in culture medium and *in vivo* tests (Sharma, 1981; Shah et al., 1985; Lenka and Ram, 1997). However, the complexity of BR prevention and control means most of these studies remain in the laboratory stage and chemical pesticides are less frequently used in practical production (Langston and Cummings, 2003; Lamichhane et al., 2017). The chemical pesticides currently in use not only pollute the environment, but also easily cause *Xcc* to form chemical resistance because of their small number. Therefore, pesticides should be used alternately in the field application process (seed soaking, leaf spraying, root irrigation) to improve the effectiveness of BR control. At the same time, new pesticides with low toxicity and high control efficiency should be developed. In addition to chemical pesticides, research on disease resistance without environmental pollution should mainly focus on the use of hot water or chemical agents for seed disinfection, the use of chemical agents or fertilizers to improve plant resistance, and the use of *Xcc* antagonism (Figure 1).

As a simple and efficient control method, physical control can effectively inhibit *Xcc* infection. The maximum temperature usually tolerated by *Xcc* is 40°C; therefore, *Xcc* pathogens can be eliminated by soaking the seeds in 50–52°C hot water, or *Xcc*-contaminated seeds can be sterilized using appropriate doses of ultraviolet light (Shah et al., 1985; Brown et al., 2001; Van Der Wolf and Van Der Zouwen, 2010; Velasco et al., 2013). *Xcc* can also be prevented by entering the plant via stomata or wounds on plant leaves by forming biofilms on the surface of plant leaves (Velasco et al., 2013; Cao et al., 2020; Pecenka et al., 2021). In addition, sterilizing nanomaterials can also be attached to the leaf surface, resulting in the death of *Xcc* in the plant via its inability to metabolize energy (Gan et al., 2010; Afrin et al., 2018; Pecenka et al., 2021). Although there are relatively few physical control methods at present, they have the characteristics of simple operation and lack of pollution; therefore, it is believed that more physical methods of BR control, especially nanomaterials, will be developed and used in Brassicaceae vegetable production in the future. In addition, some chemicals can be used for seed soaking or leaf spraying because of their sterilizing activity (Patikarnmonthon et al., 2010; Sakudo et al., 2020). Certain exogenous chemicals have similar functions to hormones, which can improve the resistance of plants to *Xcc* by enhancing their antioxidant capacity and improving their disease-resistant secondary metabolites; other chemicals can reduce the harm caused by *Xcc* by affecting its membrane stability (Qian et al., 2006; Baranek et al., 2021).

Compared with physical control and chemical control, there are more studies and reports on biological control, which suggests that researchers are more inclined to use environmentally sound methods for BR control. In the future, the potential biological control of BR will mainly focus on the use of plant extracts and *Xcc* antagonism. Compared with chemical agents, plant extracts have the advantages of a wide range of sources and less environmental pollution. A large number of crop extracts from *Acacia*, *Tylophora*, *Ocimum*, and other genera have been reported to show a controlling effect on *Xcc* (Tiwari et al., 2004; Sain, 2007; Bhardwaj and Laura, 2009; Kumar et al., 2018; Fontana et al., 2021; Table 1). Moreover, the antimicrobial activities of extracts from different parts of the same plant are also different (Bhardwaj and Laura, 2009; Bhardwaj and Bhardwaj, 2011; Table 4). The components of these plant extracts are complex; therefore, it is difficult to determine the exact substances or particular components of a class of substances that exert the antibacterial activities of the extracts. If the effective antibacterial substances cannot be determined, the study of its antibacterial mechanism becomes impossible. It is hoped that with developments in HPLC, HPLC-MS, and GC-MS technology, we can better identify the specific *Xcc* resistant substances and components in these extracts. A study showed that thymol and carvacrol from *Lippia gracilis* essential oils could control BR (Rafael et al., 2019). Therefore, the effective thymol and carvacrol could be extracted from *L. gracilis* and used as biopesticides, or could be chemically synthesized and used in Brassicaceae vegetable production. In addition, the glucosinolates and phenolics in Brassicaceae vegetables are closely associated with BR disease resistance, especially the gluconapin and its metabolite gluconapin-isothiocyanate (Aires et al., 2011; Velasco et al., 2013). Therefore, we can reduce the loss caused by BR by planting varieties with relatively high contents of *Xcc*-resistant glucosinolate and phenolics in production. Meanwhile, the glucosinolate content in Brassicaceae vegetables can be improved by regulating Gluconapin and other BR resistance glucosinolate biosynthesis pathways through genetic engineering, thereby improving resistance to BR.

**TABLE 4:** *Xcc* inhibitory components in different plant extracts.

Plant species	Part used	Solvent	Composition	Concentration	Zone of minimum inhibition (mm)*	References
*Brassica rapa*	Leaf	Methanolic	Gluconapin, gluconapin-ITC	0.150 g leaf/4 ml 70% methanolic	11.76, 8.40	Velasco et al., 2013
*Lippia gracilis*	Leaf	Water	Thymol, carvacrol	–	–	Rafael et al., 2019
*Piper hispidum*	Leaf	Water	Essential oil (trans-a-bisabolene, b-pinene, a-pinene, allo-aromadendrene, (-) spatulenol and L-linalool)	–	–	Sánchez Pérez et al., 2014

*Means items are not mentioned in the references.

Among the methods developed to control BR, the use of *Xcc*-antagonists is the most convenient and environmentally friendly method reported so far. In addition to inhibiting *Xcc* and improving crop growth index and yield, soil beneficial bacteria such as *Bacillus*, *Pseudomonas*, and other PGPRs can also improve soil microbial community structure and increase the abundance of beneficial bacteria in soil (Sain, 2007; Wulff et al., 2010; Liu et al., 2016a; Ha et al., 2017; Li’aini et al., 2017; Holtappels et al., 2022; Tables 2, 3). Crop species and soil structure also affect the control effect of PGPR genera; thus, microbial agents suitable for different crops and soil types can be developed. During planting, growers can apply the best microbial agents according to the soil characteristics of crops and planting environment. Combinations of physical, chemical, and biological control methods could also be used to reduce chemical resistance and adaptability on the premise of improving the effectiveness of BR control.

With the development of biotechnology, chemical synthesis, plant extract separation technology, and other technologies, more BR resistant varieties and efficient and environmentally friendly disease-resistance methods will be developed for use in production to reduce the losses caused by *Xcc*.

## Author contributions

ZCL and JY designed the manuscript. BX, BZ, ZZL, and ZY downloaded and summarized the references. ZCL, HW, and JW completed the manuscript. JL, SL, and SW revised the manuscript. All authors contributed to the article and approved the submitted version.
